# Particulate Matter in Second-Hand Smoke Emitted from Different Cigarette Sizes and Types of the Brand Vogue Mainly Smoked by Women

**DOI:** 10.3390/ijerph13080799

**Published:** 2016-08-08

**Authors:** Nora Kant, Ruth Müller, Markus Braun, Alexander Gerber, David Groneberg

**Affiliations:** Institute of Occupational Medicine, Social Medicine and Environmental Medicine, Goethe-University, Theodor-Stern-Kai 7, Haus 9b, Frankfurt am Main 60590, Germany; ruth.mueller@med.uni-frankfurt.de (R.M.); m.braun@med.uni-frankfurt.de (M.B.); gerber@med.uni-frankfurt.de (A.G.); groneberg@med.uni-frankfurt.de (D.G.)

**Keywords:** particulate matter, size of cigarettes, environmental tobacco smoke, menthol, automatic environmental tobacco smoke emitter

## Abstract

Indoor air pollution with harmful particulate matter (PM) is mainly caused by cigarette smoke. Super-Slim-Size-Cigarettes (SSL) are considered a less harmful alternative to King-Size-Cigarettes (KSC) due to longer filters and relatively low contents. We ask if “Combined Mainstream and Sidestream Smoke” (CMSS)-associated PM levels of SSL are lower than of KSC and thus are potentially less harmful. PM concentrations in CMSS (PM_10_, PM_2.5_, and PM_1_) are measured from four cigarette types of the brand *Vogue*, using an “automatic-environmental-tobacco-smoke-emitter” (AETSE) and laser aerosol spectrometry: SSL-BLEUE, -MENTHE, -LILAS and KSC-La Cigarette and -3R4F reference. This analysis shows that SSL MENTHE emitted the highest amount of PM, and KSC-La Cigarette the lowest. 3R4F reference emitted PM in the middle range, exceeding SSL BLEUE and falling slightly below SSL LILAS. It emerged that PM_1_ constituted the biggest proportion of PM emission. The outcome shows significant type-specific differences for emitted PM concentrations. Our results indicate that SSL are potentially more harmful for passive smokers than the respective KSC. However, this study cannot give precise statements about the general influence of the size of a cigarette on PM. Alarming is that PM_1_ is responsible for the biggest proportion of PM pollution, since smaller particles cause more harmful effects.

## 1. Introduction

In the last century, about 100 million people were killed by the use of tobacco. Tobacco related deaths will increase to 1 billion in the 21st century if smoking behavior does not change [1]. It is well known that tobacco use is highly dangerous, as “the most important risk factor for premature death in men and the second most important risk factor in women” [1]. Half of the one billion smokers will be killed by the use of tobacco, about 5 million people each year. In addition to those people who die directly because of the use of tobacco, there are another nearly 1 million people who die because of inhaling environmental tobacco smoke, or “Combined Mainstream and Sidestream Smoke” (CMSS) [2]. 

In 2014, one billion of the 7.28 billion people worldwide were smokers [2,3]. In developing countries, approximately every second man and less than 1 out of 10 women are smokers. In developed countries, the prevalence of smoking women is higher than in developing countries, and approximately 1 out of 4 men and women smoke [1,4,5]. In the last century, the number of smoking women has risen dramatically [6]. Since this time, tobacco companies even focus on women with special cigarettes—e.g., super slim size cigarettes (SSL) [7,8].

Vogue cigarettes belong to the British American Tobacco Group, one of the largest tobacco groups worldwide [1]. Vogue focuses especially on women with their design, as Vogue produces inter alia SSL, which are mainly smoked by women [9]. Vogue SSL consists of an extra-long filter. The cigarettes are 100 mm long, including a 30 mm filter and a rod circumference of 18.85 mm—much longer and thinner than usual king size cigarettes.

The main risks of smoking are well known, but in the last few years, the question of particulate matter (PM) came up. PM consists of extremely small particles distributed in the air, and may have an extremely harmful effect on human health [10]. It is well studied that PM can cause premature death, cardiovascular damage, decreased lung function, and respiratory infections and diseases [11,12,13,14]. The smaller the PM, the more damaging are the effects on health [15]. With this knowledge, CMSS is increasingly harmful to everybody’s health, as cigarette smoke produces a large quantity of PM [16,17,18].

PM is a mixture of solid particles and/or liquid droplets smaller than 10 micrometers that are inhalable by the lung. PM contains chemicals of dust or smoke. PM can be subdivided into primary particles and secondary particles. While primary particles are directly produced by a source like fire, secondary particles are the result of chemical and physical reactions emitted, for example, by cars. Those secondary particles are the main cause for particle pollution in the air, which is smaller than 10 micrometers [15,19]. 

There are three different groups of PM, which are all inhalable into our lungs. According to the United States Environmental Protection Agency (EPA) in 2013, PM is differentiated based on its size into PM_10_ (coarse) and PM_2.5_ (fine), and an extended subdivision is additionally the ultrafine PM_1_ [15,20]. PM_10_ contains particles between 2.5 µm and 10 µm, which are inhalable coarse particles. A size of 2.5 µm and smaller are called PM_2.5_, or fine particles [15]. In order to divide PM in more detail, there is a subclass called PM_1_ or ultra-fine particle, which are smaller than 0.1 µm [21]. However, there are sources which define PM_1_ as smaller than 1 µm, like it is used in the present study [22]. 

In previous studies, it became apparent that cigarette PM levels vary within different brands and types of cigarettes [16,17,23,24]. Tar content, different additives, and filter types might also have an influence on PM, so this needs to be analyzed more intensively by directly comparing different types of cigarettes from one brand with each other [25,26]. This study wants to show the PM levels of different sizes and types of Vogue cigarettes—a cigarette brand mainly smoked by women, as the number of smoking women is still rising. Vogue tobacco products with super slim size (SSL) will be compared with Vogue king-sized cigarettes (KSC) in a standardized way, and with the 3R4F reference cigarette (KSC). The different types of Vogue cigarettes are further distinguished by their tar content; one has the flavor of menthol as an additive, and one is supposed to be without additives.

## 2. Materials and Methods 

### 2.1. Tobacco Products

**Figure 1:**
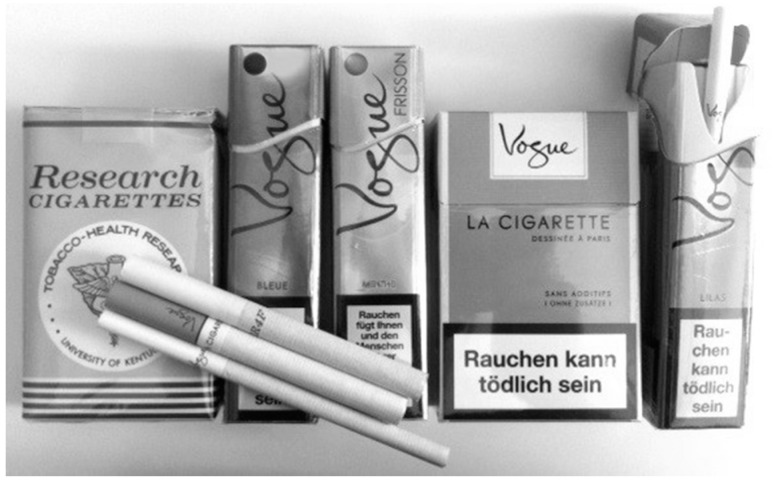
Design of the cigarettes and cigarette packets of the investigated cigarettes.

This study analyzed four types of Vogue cigarettes—Vogue La Cigarette king size cigarettes (KSC) without additives, Vogue BLEUE super slim cigarettes (SSL), Vogue MENTHE SSL with menthol as an additive, and Vogue LILAS SSL cigarettes, with the 3R4F cigarette as reference. In total, 100 cigarettes were measured: 20 3R4F cigarettes, 20 Vogue La Cigarette cigarettes, 20 Vogue BLEUE cigarettes, 20 Vogue MENTHE cigarettes, and 20 Vogue LILAS cigarettes. Vogue SSL were much longer and thinner than king size cigarettes, and with its label design Vogue had focused mostly on women (see Figure 1 and Table 1). To compare this study with studies from the literature, the standardized 3R4F reference cigarettes (College of Agriculture, University of Kentucky, USA)—manufactured for research purposes only—were used. Manufacturers’ information about tar, nicotine, and carbon monoxide, and self-measured dimensions of all investigated cigarettes are shown in Table 1.

### 2.2. Automatic Environmental Tobacco Smoke Emitter (AETSE)

Tobacco products were smoked on a standardized and reproducible cycle in a defined chamber using an automatic environmental tobacco smoke emitter (AETSE). The AETSE enabled the measurement of CMSS-PM concentrations without exposing test persons and the investigator to the dangers of tobacco smoke. Using a defined and separated room, measurement could take place under standardized closed-door conditions. The AETSE was developed and constructed by Schimpf-Ing Trondheim Norway for the needs of previous analysis, as part of the ToPIQ-II study to automatically generate CMSS in a standardized way [16,17,24]. The AETSE consisted of a 200 mL glass syringe, which generated a puff of 40 mL by pushing and pulling. The 40 mL suction volume, the amount of eight puffs needed to smoke the cigarettes, and the sequence of puffs and breaks were adjusted via a microcontroller that belonged to the AETSE. A double-puff was needed to light the cigarette; otherwise, the cigarette would extinguish too easily. Every puff took 3 s, followed by a break of 24 s, in which the cigarette smoldered, followed by another puff of 3 s, and so forth. Automatic smoking was possible via a stepper motor. Detailed information about the AETSE can be found in the ToPIQ-II study [17]. This procedure imitated the puffs and exhalations of a smoker, which was defined as mainstream smoke (MS). Between each puff, sidestream smoke (SS) was constantly produced by the burning of the cigarette, and together with the MS, CMSS was created. 

### 2.3. Measurement Equipment

To measure the PM_10_, PM_2.5_, and PM_1_ concentrations in the glass chamber, a portable laser aerosol spectrometer (Model 1.109, Grimm Co., Ainring, Germany) was used. The aerosol spectrometer measured airborne particles using light scattering. PM_10_ concentrations measured by Grimm Aerosol Technik GmbH & Co. KG (2012) [22] included all particles with a size greater than 250 nm and less than or equal to 10 µm, because at this size, the measurement equipment reached its limit [27]. This limitation excluded particles in the PM_2.5_ range, as PM_2.5_ extends to 0.1 µm. However, it still enables the measurement of the biggest proportion of PM_2.5_ particles (93.75%). However, with this measurement equipment, PM_10_ was analyzed without the limitation that particle size must be larger than 2.5 µm. Accordingly, PM_10_ was defined in this study to be >250 nm and ≤10 µm. PM_2.5_ included all particles >250 nm and ≤2.5 µm, and PM_1_ included all particles >250 nm and ≤1 µm. In contrast to PM classification by its actual definition according to EPA (2013) [12], this study uses PM_10_ concentrations which also include PM_2.5_ and PM_1_, and PM_2.5_ concentrations also include PM_1_. To display PM_10_ concentrations by their actual definition, first PM_2.5_ concentrations need to be subtracted. Accordingly, to display PM_2.5_ concentrations by their actual definition, first PM_1_ concentrations need to be subtracted. This enabled the measurement of particles with a minimum size of 0.25 µm, and classified them into a definite particle size. AETSE ran volume controlled, with a volume flow-rate of 1.2 L·min^−1^, and PM concentrations were measured every 6 s [28]. As tobacco smoke produced heavy soiling (such as sticky products and damage from tar) which would cover the measurement equipment, the aerosol spectrometer was placed outside the glass chamber at the backside on a board. A 15 cm suction hose connected the inside, where sample air was sucked, with the outside, where the sample air was measured. In addition, sample air was diluted pre-analytically in a dilution ratio of 1:10 using neutral compressed air via a dilution system VKL mini (Model 7.951, Grimm Co., Ainring, Germany) that was connected to the suction hose. As the glass chamber is a separate laboratory room and was cleaned before and after every cycle of measurement, daily variable environmental PM concentrations did not exert an influence on the measurements. Before every cycle started, baseline PM concentrations inside the glass chamber were checked to be approximately 0.5 µg·m^−3^, with dilution for all PM values. 

### 2.4. Smoking Protocol

In the previous ToPIQ-II study, the basis for this smoking protocol was developed [17]. The smoking protocol cycle consisted of four phases: (1) a 5 min pre-igniting phase; (2) a 4 min and 55 s combustion phase; (3) a 5 min post-combustion phase; and (4) a 5 min suction phase. In this way, 150 values were received per cigarette. 

The combustion phase was adapted to the Vogue SSL cigarettes. To smoke as many puffs as possible without smoking closer than 0.5 cm to the filter, eight identical puffs of 40 mL puff volume were needed. To receive reproducible data, 20 cigarettes of each brand were smoked with this standardized smoking protocol. The cigarette was smoked following this precise protocol, while measuring the PM levels every 6 s.

### 2.5. Data Processing

PM_10_, PM_2.5_, and PM_1_ levels were analyzed using the area under the curve (AUC) and mean concentration (C_mean_) in a dilution ratio of 1:10. The dilution ratio was finally back-converted. The AUC describes the exposure 5 min before, during, and 5 min after smoking a cigarette in this study. C_mean_ of PM levels were summarized at the end to enable comparison with other scientific studies, which mostly use C_mean_ to demonstrate particulate concentrations.

Regarding the AUC, a test for artificial peaks was done in order to estimate the reliability of single measurements. To indicate extreme impacts of mainstream smoke, artificial peaks were defined to be greater than threefold of the AUC values measured during the post-combustion phase. Five random smoking cycles of each type of cigarette were analyzed. The AUC including artificial peaks was compared to the AUC excluding artificial peaks. The proportion of artificial peaks from the total AUC including artificial peaks was in general less than 17% (the internal limit was set for 22%). This value below our internal limit should minimize technical errors responsible for the extreme impact of mainstream smoke. 

The AUC and C_mean_ values per cigarette type were tested for outliers (Grubbs’ test) using a significance level of 0.05 [29]. Within the 3R4F reference cigarettes, one outlier was detected in the PM_2.5_ and PM_10_ results. In both cases, it was the same cigarette. Within the Vogue BLEUE cigarettes, one outlier was detected for PM_1_, one for PM_2.5_, and one for PM_10_ (three cigarettes). In the Vogue MENTHE, Vogue La Cigarette, and Vogue LILAS groups, no significant outlier was detected.

Afterwards, a test for Gaussian normality of the revised AUC and C_mean_ was done using the D'Agostino-Pearson test with a significance level of *p* > 0.05 [30]. PM_10_, PM_2.5_, and PM_1_ demonstrated Gaussian distributions for each tested cigarette type, except for Vogue LILAS PM_1_ (*p* = 0.04). Here, the normal distribution was found with the Kolmorov–Smirnov-test (*p* = 0.07). To see if there are any significant differences between the PM levels of the different types of Vogue cigarettes, an ANOVA including a Tukey post-test was done by comparing the different types of Vogue cigarettes among themselves. The ANOVA assumption that variances are equal across groups was verified with the Bartlett test at the level of *p* = 0.01.

## 3. Results

The brand-specific PM values (C_mean_, AUC) for three PM classes are listed in Table 2. The AUC and C_mean_ values of each investigated cigarette are in relation equal to each other, as the AUC is dependent on the time required to smoke a cigarette, and eight puffs (and thus the same time) were required to smoke all investigated cigarettes. This means that the percentage differences of AUC-PM and C_mean_ of all investigated cigarettes are the same.

**Figure 2:**
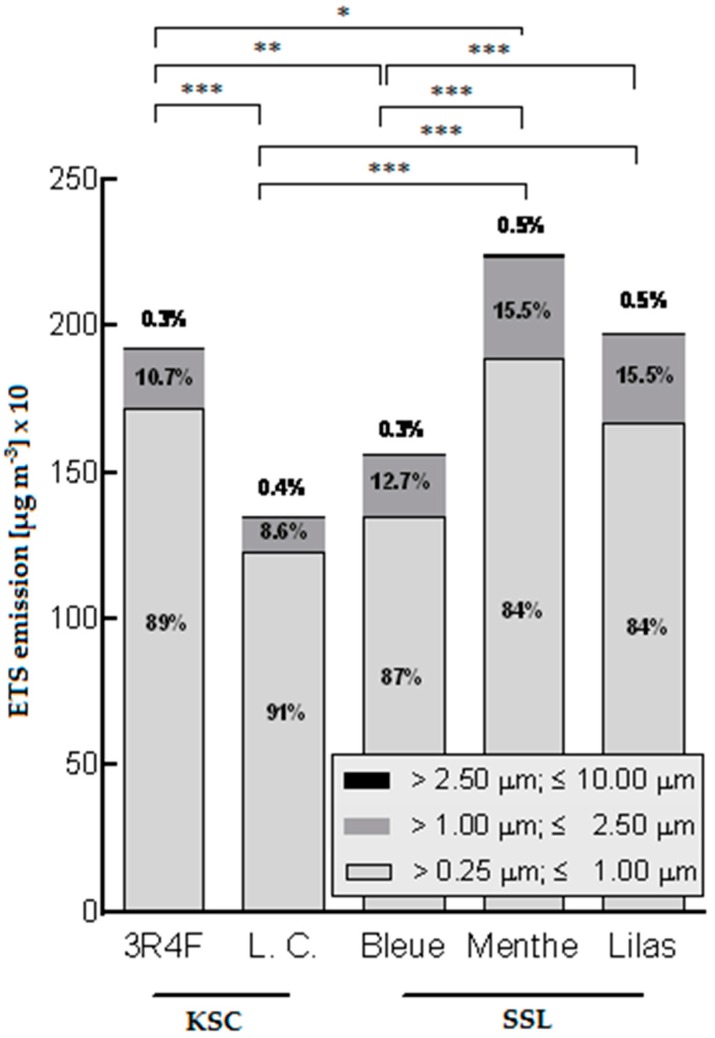
Distribution pattern of PM emissions in CMSS and comparison of KSC 3R4F (reference), KSC Vogue La Cigarette (L.C.), and SSL cigarettes (Bleue, Menthe, Lilas). Stars represent significance level of post hoc test with * *p* < 0.05, ** *p* < 0.01, and *** *p* < 0.001. KSC—king size cigarette, SSL—super slim.

In general, KSC and SSL reveal varying amounts of PM in CMSS, as deduced from the comparative analysis between the two types of KSC, 3R4F reference cigarette and Vogue La Cigarette without additives, and the three types of SSL, Vogue BLEUE, Vogue MENTHE with menthol as an additive, and Vogue LILAS (see Figure 2). Interestingly, the highest amount of PM emissions was measured for the SSL Vogue MENTHE, which emitted 17% higher PM_10_, 16% higher PM_2.5_, and 10% higher PM_1_ compared with the reference cigarette. The lowest amount of PM emissions was found for the KSC Vogue La Cigarette without additives, which revealed 30% lower PM_10_ and PM_2.5_, and 29% lower PM_1_ emissions compared with the reference cigarette. 

Figure 2 shows inter alia a comparison between the different Vogue cigarettes. The KSC Vogue La Cigarette without additives have 9%–13% less PM-C_mean_ values than Vogue BLEUE SSL, which is not significant (*p* > 0.05). Vogue MENTHE cigarettes emit 35%–40% more PM in CMSS than Vogue La Cigarette. Further, Vogue LILAS cigarettes emit 26%–32% more PM in CMSS than Vogue La Cigarette. Vogue LILAS have 23%–27% higher PM-C_mean_ values than Vogue BLEUE. Vogue MENTHE cigarettes have 40%–44% higher PM-C_mean_ values than Vogue BLEUE. Furthermore, Vogue MENTHE cigarettes with menthol as an additive emit higher amounts of PM than Vogue LILAS cigarettes, even if the difference is not significant.

PM concentrations subdivided by size (Figure 2) reveal that PM is mainly composed by PM_1_ (84%–91%), whereas PM_2.5_ emissions (8.6%–15.5%) and PM_10_ emissions (0.3%–0.5%) represent only a small proportion of CMSS associated PM. 

## 4. Discussion

Super Slime Size (SSL) cigarettes are considered a less harmful alternative to king size cigarettes (KSC), due to their longer filter and relatively low CO, tar, and nicotine contents. Our comparison of CMSS-associated PM levels of Vogue-brand SSL cigarettes of with KSCs (Vogue, 3R4F) revealed that SSL-associated PM levels are even higher than in Vogue-brand KSC. Thus, our results indicate that Vogue-brand SSL are potentially more harmful for passive smokers than the respective KSC. Furthermore, all cigarettes emit a high percentage of mainly low-sized PM (<1 µm), which is considered as respirable and hence potentially health harming for passive smokers [17,31]. 

A non-standard smoking regime was used to acquire PM emissions in CMSS; therefore, data from this study cannot be compared to studies using a standard smoking regime. However, the aim was to generate relative data in terms of comparing Vogue cigarettes with the standard research cigarette 3R4F regarding different PM emissions. 

No significant difference can be seen when comparing the KSC Vogue La Cigarette with SSL Vogue BLEUE, which have the same content of tar and nearly the same amount of nicotine. In addition, SSL Vogue BLEUE cigarettes were compared with SSL Vogue LILAS cigarettes, as they differ in their content of tar and nicotine. It is now surprising that even though SSL Vogue BLEUE cigarettes contain more tar and nicotine, they emit significantly lower levels of PM in CMSS than SSL Vogue LILAS cigarettes. Vogue LILAS cigarettes have 27% higher PM_10_ and PM_2.5_ emissions and 23% higher PM_1_ emissions than Vogue BLEUE. This result reveals that tar and nicotine are not indicative of the amount of CMSS-associated PM.

A significant difference can be seen when comparing KSC Vogue La Cigarette with SSL Vogue LILAS. SSL Vogue LILAS emitted 26%–32% more PM in CMSS than the KSC. As SSL Vogue LILAS have lower amounts of tar and nicotine than KSC Vogue La Cigarette, these results could make up the case that Vogue SSL cigarettes emit higher amounts of PM in CMSS than KSCs. However, KSC Vogue La Cigarette are supposed to have no additives. Additives are a major factor for the health harming effects of cigarettes, because they are made up of many carcinogenic and toxic chemicals—for example, to make smoke more easily inhalable and to generate different flavors [25,32]. Therefore, it might be comprehensible why these cigarettes without additives have by far the lowest amounts of PM.

Comparing SSL Vogue MENTHE with menthol and SSL Vogue BLEUE enables direct comparison in relation to menthol as an additive, as both types of cigarettes are SSL with the same amount of tar and nicotine. It emerged that Vogue MENTHE has by far higher PM emissions than Vogue BLEUE (40% for PM_1_, 44% for PM_10_ and PM_2.5_). These differences can only be comprehensible if the menthol additive in Vogue MENTHE emits a lot of PM. Menthol as an additive has harmful effects on smokers because it enables cigarette smoke to be more easily inhaled [25,33]. Furthermore, this study indicates another harmful effect of menthol as an additive, because it apparently severely increases PM levels, which are harmful for the health of active as well as passive smokers. An increase of PM emissions caused by higher amounts of additives was also shown by Rustemeier et al. (2002) in a previous study [26]. From this, it can be concluded that menthol as an additive in Vogue cigarettes may increase the PM concentrations emitted from CMSS, and the absence of any additives in Vogue cigarettes decreases the PM emissions. However, this study cannot give precise statements about the influence of additives in cigarettes on PM concentrations emitted in CMSS.

In order to investigate the PM pollution caused by CMSS, PM_10_, PM_2.5_, and PM_1_ concentrations were used as C_mean_ and AUC in this study. PM_2.5_ is considered the standard measurement to investigate PM concentrations in the air, but PM_10_ is also often used [15,34]. However, this study also investigated PM_1_ concentrations, since this value is probably attributed to more importance in the future, according to a study by Meng et al. (2013) stating that smaller particles have more harmful effects on human health [31]. There is a great number of studies, including a WHO report (2004) about the health effects of high PM concentrations [14]. Based on that, statements can be made about the potential health effects of cigarettes used in this study, related to their amounts of emitted PM, as CMSS results in an increase of PM concentration [16,17,18]. We can assume that cigarettes with higher emitted PM concentrations have more damaging impact on smokers and passive smokers, such as premature death, cardiovascular damage, decreased lung function, and respiratory infections and diseases [11,12,13,14]. The increase of PM concentrations caused by CMSS is also impressively shown in the present study, especially for SSL Vogue MENTHE with menthol as an additive and SSL Vogue LILAS, who emit by far the highest values of PM. It is alarming that PM_1_ is reasonable for the biggest proportion of PM pollution, since smaller particles can reach deeper into the respiratory tract and have more harmful effects on human health [31]. 

As this study is an experimental study about PM concentrations emitted from cigarettes smoked in a separate room, no final statements about health hazard effects for passive smokers can be made. The AETSE is a tobacco smoke emitter, which smokes a cigarette automatically by sucking cigarette smoke into a glass syringe and blowing it out again according to defined instructions. Originally, it was developed and constructed by Schimpf-Ing Trondheim Norway for the needs of previous analysis to generate CMSS automatically in a standardized way [17,24]. Although test smokers would guarantee more realistic smoking situations and emissions, their exposure to harmful tobacco smoke would not be acceptable. For occupational health and safety reasons, the AETSE was constructed to receive reliable and reproducible data to compare PM-amounts between different brands and types of cigarettes without exposing test smokers to harmful tobacco smoke. In real life, PM levels can vary a lot, as every smoker has a different habit of smoking, resulting in different durations of drags and breaks, and different puff volume [35]. Longer drag duration produces more MS, and longer breaks in between two drags produces more SS. However, these variances are trivial as long as the aim is to compare PM levels of different types of cigarettes with each other, and for this specific reason the standardized smoking protocol was developed.

It is unambiguous that differences in CMSS-PM emissions exist between different types of cigarettes, especially between the SSL. These results need further investigation to better understand why PM levels differ between different brands and types of cigarettes. It is worth to investigating which determinants increase PM levels to understand why some cigarettes are potentially more harmful for passive smokers than others.

## 5. Conclusions 

The present study pointed out that there are significant differences in PM concentrations between different types of cigarettes, and that PM_1_ is responsible for the main part of CMSS-associated PM exposure. So far, the exact cause of these differences is still unclear. The present study, as well as previous studies, imply that content of tar, the type and length of cigarette (KSC, SSL), and different additives may influence PM emissions. Menthol seems to increase PM concentrations, and cigarettes without additives seem to decrease them. This study cannot ultimately answer the question of whether SSL emit lower or higher levels of PM than KSC in general, so this should be investigated more precisely. Further research of other brands and types of cigarettes is necessary to understand the origin of PM in CMSS.

## Figures and Tables

**Table 1:** Characteristics of KSC 3R4F (reference), KSC Vogue La Cigarette, and three SSL cigarettes (Bleue, Menthe, Lilas). KSC—king size cigarette, SSL—super slim size.

	3R4F	Vogue La Cigarette	Vogue BLEUE	Vogue MENTHE	Vogue LILAS
Tar (mg)	9.50	6.00	6.00	6.00	4.00
Nicotine (mg)	0.73	0.50	0.60	0.60	0.40
Carbon monoxide (mg)	12.00	7.00	4.00	4.00	3.00
Cigarette Length (mm)	84.00	83.00	100.00	100.00	100.00
Tobacco Rod Circumference (mm)	24.80	24.82	18.85	18.85	18.85
Tobacco Rod Length (mm)	57.00	57.00	70.00	70.00	70.00
Filter Length (mm)	27.00	25.00	30.00	30.00	30.00
Tobacco volume (cm^3^)	2.79	2.79	1.98	1.98	1.98
Cigarette Weight (g)	1.05	0.78	0.60	0.58	0.56
Tobacco Weight (g)	0.78	0.55	0.43	0.42	0.40
Filter Weight (g)	0.28	0.19	0.12	0.12	0.12
Filling density (g·cm^−3^)	0.28	0.20	0.30	0.29	0.28

**Table 2:** Comparative table of particulate matter (PM) PM_10_, PM_2.5_, and PM_1_ emissions in the combined mainstream and sidestream smoke (CMSS) of KSC 3R4F (reference), KSC Vogue La Cigarette, and three SSL cigarettes (Bleue, Menthe, Lilas) on the basis of area under the curve (AUC) and C_mean_.

	PM_10_		PM_2.5_		PM_1_	
Cigarette	AUC (mg·m^−^³·s^−1^)	C_mean_ (µg·m^−^³)	AUC (mg·m^−^³·s^−1^)	C_mean_ (µg·m^−^³)	AUC (mg·m^−^³·s^−1^)	C_mean_ (µg·m^−^³)
3R4F reference	1724 ± 62	1926 ± 69	1719 ± 61	1920 ± 68	1541 ± 44	1721 ± 49
Vogue La Cigarette	1208 ± 74	1349 ± 83	1204 ± 74	1344 ± 82	1100 ± 58	1229 ± 64
Vogue BLEUE	1395 ± 46	1559 ± 51	1390 ± 45	1555 ± 50	1208 ± 30	1350 ± 33
Vogue MENTHE	2010 ± 76	2224 ± 85	2000 ± 75	2233 ± 84	1694 ± 60	1892 ± 67
Vogue LILAS	1768 ± 63	1974 ± 71	1761 ± 62	1965 ± 69	1492 ± 43	1666 ± 48

## References

[1] Eriksen M., Mackay J. (2015). The Tobacco Atlas. http://www.tobaccoatlas.org.

[2] WHO (2015). Tobacco. http://www.who.int/mediacentre/factsheets/fs339/en/.

[3] Stiftung Weltbevölkerung (2014). Zum Jahreswechsel Leben 7.284.283.000 Menschen auf der Erde. http://www.weltbevoelkerung.de/aktuelles/details/show/details/news/zum-jahreswechsel-leben-7284283000-menschen-auf-der-erde.html.

[4] Chollat-Traquet C.M., WHO (1992). Women and Tobacco.

[5] Office of National Statistics (2000). Social Trends 30.

[6] Doll R., Derby S., Whitley E., Charlton J. (1997). Trends in mortality from smoking—related diseases. The Health of Adult Britain: 1841–1994.

[7] Amos A., Haglund M. (2000). From social taboo to “torch of freedom”: The marketing of cigarettes to women. Tob. Control.

[8] British American Tobacco International Our Brands. http://www.bati.com/group/sites/BAT_8F3HTL.nsf/vwPagesWebLive/DO8F3HZZ?opendocument.

[9] Samet J.M., Yoon S. (2010). Gender, Women, and the Tobacco Epidemic: Why Women and Girls Use Tobacco.

[10] AIR Info Now What Is Particulate Matter?. http://www.airinfonow.org/html/ed_particulate.html.

[11] Dockery D.W., Pope C.A., Xu X.P., Spengler J.D., Ware J.H., Fay M.E. (1993). An association between air-pollution and mortality in 6 united-states cities. N. Engl. J. Med..

[12] EPA (2014). Particulate Matter (PM). http://www.epa.gov/airquality/particlepollution/health.html.

[13] Gordian M.E., Ozkaynak H., Xue J.P., Morris S.S., Spengler J.D. (1996). Particulate air pollution and respiratory disease in Anchorage, Alaska. Environ. Health Persp..

[14] WHO (2004). Health Aspects of Air Pollution: Results from the WHO Project “Systematic Review of Health Aspects of Air Pollution in Europe”.

[15] EPA (2013). Particulate Matter. http://www.epa.gov/pm/basic.html.

[16] Gerber A., Bigelow A., Schulze M., Groneberg D.A. (2015). Brand cigarillos—A cheap and less harmful alternative to cigarettes? Particulate matter emissions suggest otherwise. Int. J. Environ. Res. Public Health.

[17] Gerber A., Hofen-Hohloch A.V., Schulze J., Groneberg D.A. (2015). Tobacco smoke particles and indoor air quality (ToPIQ-II)—A modified study protocol and first results. J. Occup. Med. Toxicol..

[18] Schwartz J., Zeger S. (1990). Passive smoking, air pollution, and acute respiratory symptoms in a diary study of student nurses. Am. Rev. Respir. Dis..

[19] Environment Canada (2013). Particulate Matter. http://www.ec.gc.ca/air/default.asp?lang=En&amp;n=2C68B45C-1.

[20] GreenFacts (2001–2012). Air Pollution Particulate Matter. http://www.greenfacts.org/en/particulate-matter-pm/level-2/01-presentation.htm#1.

[21] Morawska L., Moore M.R., Ristovski Z.D. (2004). Health Impacts of Ultrafine Particles: Desktop Literature Review and Analysis.

[22] Grimm Aerosol Technik GmbH & Co. KG (2012). Grimm Software für Optical Particle Counter: Tragbares Aerosolspektrometer 1.108/1.109.

[23] Müller D., Schulze J., Ackerman H., Klingelhoefer D., Uibel S., Groneberg D.A. (2012). Particulate matter (PM) 2.5 levels in ETS emissions of a Marlboro Red cigarette in comparison to the 3R4F reference cigarette under open- and closed-door condition. J. Occup. Med. Toxicol..

[24] Wasel J., Boll M., Schulze M., Mueller D., Bundschuh M., Groneberg D.A., Gerber A. (2015). Brand cigarillos: Low price but high particulate matter levels—Is their favorable taxation in the European Union justified?. Int. J. Environ. Res. Public Health.

[25] Geiss O., Kotzias D. (2007). Tobacco, Cigarettes and Cigarette Smoke: An Overview.

[26] Rustemeier K., Stabbert R., Haussmann H.J., Roemer E., Carmines E.L. (2002). Evaluation of the potential effects of ingredients added to cigarettes: Part 2: Chemical composition of mainstream smoke. Food Chem. Toxicol..

[27] Grimm Aerosol Technik GmbH & Co. KG (2015). Indoor Air Quality Monitors. http://grimm-aerosol.de/index.php.

[28] Grimm Aerosol Technik GmbH & Co. KG (2010). Portable Laser Aerosolspectrometer and Dust Monitor: Model 1.108/1.109.

[29] GraphPad Software I (2015). QuickCalcs. http://graphpad.com/quickcalcs/grubbs1/.

[30] GraphPad Software I (2015). GraphPad Software. http://www.graphpad.com/scientific-software/prism/.

[31] Meng X., Ma Y., Chen R., Zhou Z., Chen B., Kan H. (2013). Size-Fractionated Particle Number Concentrations and Daily Mortality in a Chinese City. Environ. Health Persp..

[32] Rabinoff M., Caskey N., Rissling A., Park C. (2007). Pharmacological and Chemical Effects of Cigarette Additives. Eur. Heart J..

[33] Jacobs M. From the First to the Last Ash. http://healthliteracy.worlded.org/docs/tobacco/Tobacco.pdf.

[34] Umweltbundesamt GmbH (2015). Feinstaub (PM_2.5_). http://www.umweltbundesamt.at/umweltsituation/luft/luftschadstoffe/staub/pm25/.

[35] Hatsukami D.K., Morgan S.F., Pickens R.W., Champagne S.E. (1990). Situational factors in cigarette smoking. Addict. Behav. Rep..

